# Buprenorphine for Opioid Use Disorder in the Emergency Department: A Retrospective Chart Review

**DOI:** 10.5811/westjem.2020.6.46452

**Published:** 2020-08-24

**Authors:** Kathy T. LeSaint, Brent Klapthor, Ralph C. Wang, Curtis Geier

**Affiliations:** * University of California, San Francisco, Department of Emergency Medicine, San Francisco, California; † Kaiser Permanente San Diego Medical Center, Department of Emergency Medicine, San Diego, California; ‡ University of California, San Francisco, Department of Clinical Pharmacy, San Francisco, California

## Abstract

**Introduction:**

Emergency care providers routinely treat patients with acute presentations and sequelae of opioid use disorder. An emergency physician and pharmacist implemented a protocol using buprenorphine for the treatment of patients with opioid withdrawal at an academic, Level I trauma center. We describe our experience regarding buprenorphine implementation in the emergency department (ED), characteristics of patients who received buprenorphine, and rates of outpatient follow-up.

**Methods:**

We conducted a retrospective chart review of all patients in the ED for whom buprenorphine was administered to treat opioid withdrawal during an 18-month period from January 30, 2017–July 31, 2018. Data extraction of a priori-defined variables was recorded. We used descriptive statistics to characterize the cohort of patients.

**Results:**

A total of 77 patients were included for analysis. Thirty-three patients (43%) who received buprenorphine did not present with the chief complaint of opioid withdrawal. Most patients (74%) who received buprenorphine last used heroin, and presented in moderate opioid withdrawal. One case of precipitated withdrawal occurred after buprenorphine administration. Twenty-three (30%) patients received outpatient follow-up.

**Conclusions:**

This study underscores the safety of ED-initiated buprenorphine and that buprenorphine administration in the ED is feasible and effective.

## INTRODUCTION

As of 2017, 2.1 million Americans were suffering from opioid use disorder (OUD), a condition associated with a 20-fold increase in rates of early death.^1^–^2^ While medications with proven benefit exist for the treatment of OUD, their use has not yet become widespread.^1^,^3^,^4^ Emergency departments (ED) are a natural setting for the improvement of this care, as providers routinely treat patients with acute presentations and sequelae of OUD. Calls from the Office of the Surgeon General, the US Centers for Disease Control and Prevention, and numerous state governments have specifically suggested this be done through expanded ED use of buprenorphine.^5^–^7^

Prior investigators have shown the potential of the ED as a critical point of access for patients suffering from OUD, finding that ED-initiated medications for opioid use disorder (MOUD) is feasible, efficacious, and associated with significantly increased rates of engagement in addiction treatment.^8^ In the state of California, the California Bridge Program seeks to expand and increase access to MOUD whereby participating EDs implement protocols to treat patients with OUD and connect those patients with outpatient treatment centers for sustained MOUD.^9^ However, there remains a relative lack of formal research regarding ED-initiated MOUD protocols and especially so given the scale of the current opioid overdose epidemic.^6^,^10^ Differing approaches to the use of buprenorphine for opioid withdrawal in the ED have been proposed (eg, meeting a certain Clinical Opiate Withdrawal Scale [COWS] threshold before medication administration), but description of their use in the clinical setting has thus far been limited.^11^–^13^

The goal of this study was to describe our experience regarding implementation of a protocol using buprenorphine for patients presenting with opioid withdrawal in the ED of an academic, Level I trauma center. Specifically, we sought to describe the main adverse event associated with buprenorphine administration (precipitated withdrawal) and rates of linkage to care.

## MATERIALS AND METHODS

### No Patient and Public Involvement

This research was done without patient involvement. Patients were not invited to comment on the study design nor were any consulted to develop patient-relevant outcomes or interpret the results. Patients were not invited to contribute to the writing or editing of this document for readability or accuracy. This study was approved by the University of California, San Francisco’s institutional review board and informed consent was waived given the minimal risk to subjects involved in a retrospective review of health records.

### Setting

This retrospective cohort study was conducted at Zuckerberg San Francisco General Hospital & Trauma Center (ZSFG). ZSFG is the only public hospital for San Francisco, and is the highest volume ED in the San Francisco Bay Area. Over 73,000 patients are treated annually at ZSFG, and it is the only Level I trauma center for the city and county of San Francisco. From June 2016–May 2018, the ZSFG ED saw 633 unique patients with opioid withdrawal or OUD, although this number is likely an underestimate as only the primary diagnosis is coded by the hospital’s billing services.

### Implementation and Treatment Protocol

Addiction care for ZSFG ED patients with OUD has been growing since early 2017. In January 2017, with minimal funding and technical assistance from the California Health Care Foundation (CHCF), an emergency physician (EP) champion and a clinical pharmacist worked together to implement a protocol using buprenorphine for the treatment of patients with opioid withdrawal. The implementation was part of CHCF’s creation of a project aimed at piloting a treatment model that had previously proven successful in other hospital settings. The EP champion and pharmacist also received coaching and technical assistance from CHCF’s pilot lead. This study pre-dates the now widely known California Bridge Program, which offers formalized guidance regarding ED initiation of MOUD.^9^

**Population Health Research Capsule**

What do we already know about this issue?

Buprenorphine is an effective treatment for opioid use disorder. Description of its use in the emergency department (ED) is limited.

What was the research question?

What were the characteristics and outcomes of patients who were administered buprenorphine for opioid withdrawal in the ED?

What was the major finding of the study?

Buprenorphine was administered to 77 patients; 1% had precipitated withdrawal, and 30% received outpatient follow-up.

How does this improve population health?

Buprenorphine administration in the ED is feasible and can help optimize treatment for patients with opioid use disorder.

Prior to the initiation of ZSFG’s treatment protocol, the EP champion and clinical pharmacist performed literature reviews and had several meetings with the CHCF pilot lead to develop a thorough understanding of buprenorphine. The EP champion also met with directors of several outpatient clinics and opioid treatment programs to come to an agreement on a single outpatient site where discharged patients could follow up for continued access to buprenorphine. Approval for the protocol implementation was obtained from ED leadership and the hospital-wide Pharmacy & Therapeutics Committee. The two site leads performed teaching of the protocol to ED providers at several on-site faculty meetings, pre-shift nursing team huddles, and at residency conferences. This “start-up” period totaled approximately three months. In addition, for the first six months of the protocol implementation, the EP champion carried a 24/7 pager to provide as-needed technical assistance to all ED providers.

**Figure 1:**
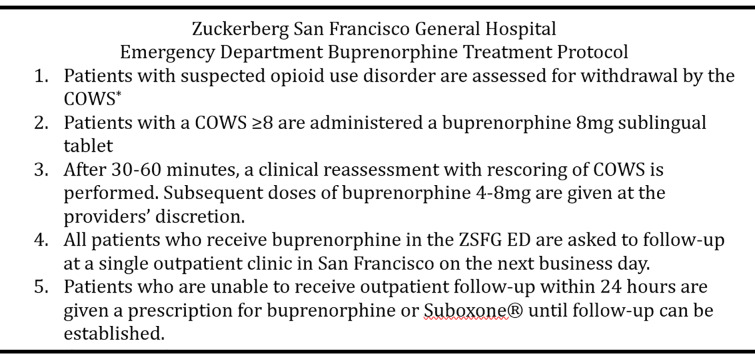
Buprenorphine treatment protocol in the emergency department at Zuckerberg San Francisco General Hospital. *Mg*, milligram; *ED*, emergency department; *ZSFG*, Zuckerberg San Francisco General Hospital.

For the first few months of the study, patients with suspected opioid withdrawal were assessed using the Short Opiate Withdrawal Scale (SOWS).^14^ After seven months, however, the protocol was revised to use COWS in an effort to standardize opioid withdrawal assessment in the ED and inpatient units.^15^ The final ZSFG protocol (Figure 1) was based on the suggested algorithm by Herring et al and as described by the current California Bridge Program.^6^, ^16^, ^17^

Patients who met the threshold for moderate withdrawal (SOWS ≥10; COWS ≥8) were administered buprenorphine (8 milligrams [mg], per protocol). Withdrawal reassessment was then performed 30–60 minutes later. Subsequent dosing of 4–8 mg of buprenorphine was given at the provider’s discretion. All patients who received buprenorphine in the ZSFG ED were given a referral for next business day follow-up at a single outpatient clinic in San Francisco. Patients who were unable to attend outpatient follow-up within 24 hours were given a prescription for buprenorphine until follow-up could be established.

### Selection of Participants

In this study, an ED pharmacist identified all patients for whom buprenorphine was ordered by a clinician via the medication administration record during an 18-month period from January 30, 2017–July 31, 2018. Patients who were not administered the ordered buprenorphine were excluded from the study. Subsequently, we surveyed electronic health records (EHR) to determine the reason buprenorphine was given. Additional patients were excluded if they were not in opioid withdrawal (eg, the patient was on chronic buprenorphine therapy and wanted a dose/refill of their medication). All patients who received buprenorphine for opioid withdrawal in the ED were included in the data analysis.

### Methods of Measurement and Data Collection and Processing

We defined all variables for data collection a priori. A researcher-made data extraction form was developed in accordance with the study objective, which included the patient’s demographic characteristics; date of service; ED length of stay; SOWS or COWS score assessments; dosages of administered buprenorphine; occurrence of precipitated withdrawal; and whether the patient followed up at the designated outpatient site within one week of ED discharge. Follow-up was tracked by reviewing patients’ EHRs for a clinic progress note, as the designated outpatient site uses the same EHR as the hospital. The study data were collected from the same medical charts by two abstractors (BK and CG). Both abstractors were hospital employees and so were well versed in the EHR. Training included reviewing 10% of all charts together with a third investigator (KL). The investigators met periodically to resolve discrepancies. The third investigator (KL) would settle any unresolved disputes by review of the specific chart.

The inter-rater reliability of two variables of interest – prevalence of precipitated withdrawal, and proportion of patients who followed up at one week – were compared for inter-rater agreement. Cohen’s kappa statistic, κ, between our abstractors for the presence of precipitated withdrawal (1.0) and outpatient follow-up (0.85) was excellent (100% and 93.4%, respectively). We collected the data in a secure onsite location and database to avoid the loss of charts and confidential information.

### Primary Data Analysis

We used descriptive statistics to characterize the cohort of patients in our study. We calculated medians and interquartile ranges (IQR) to describe the distribution of skewed numerical values such as age, while categorical variables such as race, chief complaint, and last opioid used were tabulated and reported as percentages. Data and all calculations were evaluated with Microsoft Excel 2011 (Microsoft Corporation, Redmond, WA) and STATA statistical software release 13 (StataCorp LP, College Station, TX).

## RESULTS

**Figure 2:**
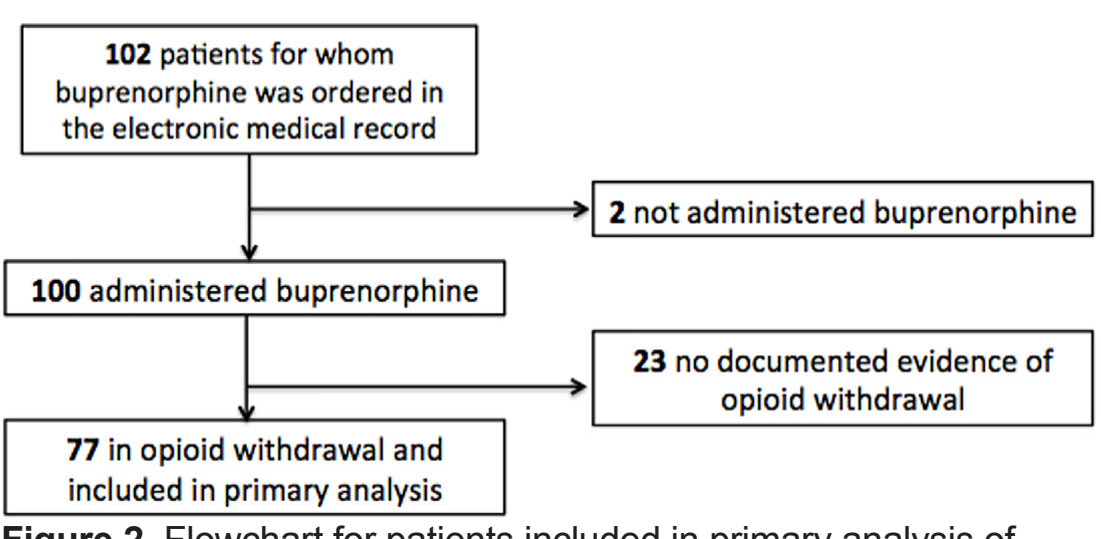
Flowchart for patients included in primary analysis of patients for whom buprenorphine was ordered in the emergency department for opioid withdrawal.

During the study period (January 30, 2017–July 31, 2018), buprenorphine was ordered for 102 ED patients. Of those, 77 patients were included for analysis (Figure 2).

Table 1 summarizes the baseline characteristics of the cohort. The median age of patients was 37 years (IQR 31–50), and 20 (26%) were female. The largest proportion of patients were White (48%), followed by Black (30%), Latino/Hispanic (20%), and Asian (1%), while race was unknown in 1% of the cohort. Thirty-three (43%) patients who received buprenorphine presented to the ED without a chief complaint of opioid withdrawal. Of these patients, 12 presented with complaints of localized pain (eg, arm, back, chest, flank, foot, knee, pelvic, tooth). Others presented with a primary psychiatric complaint such as suicidal ideation or anxiety (N = 4); after an assault (N = 3); with abscesses (N = 2;, or generalized weakness (N = 2). Other, less common, chief complaints included altered mentation, foreign body ingestion, rectal bleeding, seizure, and urinary retention.

Most patients (N = 57, 74%) who received buprenorphine in the ED last used heroin prior to being diagnosed with opioid withdrawal. Other commonly used opioids prior to presentation included buprenorphine (N = 6, 8%); methadone (N = 4, 5%); oxycodone (N = 4, 5%); and fentanyl (N = 1, 1%). For non-methadone opioids, the median time since last opioid use was 24 hours.

Table 2 details buprenorphine administration for the 77 patients in our cohort. Eighteen patients were initially assessed with the SOWS, while 41 patients had an initial COWS. Sixteen patients deemed to be in opioid withdrawal did not receive either assessment scores. There was considerable variation in practice, such as patients continuing to receive buprenorphine despite not receiving additional scoring or not meeting the set thresholds for precipitated withdrawal. However, in a majority of the cases, providers followed the protocol set in place.

One case of documented precipitated withdrawal occurred in our cohort. A 54-year-old man with a history of daily heroin insufflation presented to the ED with the chief complaint of nausea and anxiety requesting detoxification from opioids after last having used heroin four hours prior to arrival. He had never received medications for OUD in the past. His triage vital signs were as follows: blood pressure 132/91 millimeters of mercury (mm Hg), heart rate 98 beats per minute (bpm), respiratory rate 18 breaths per minute, and oxygen saturation 99% on room air. The patient was initially seen by an advanced practice provider in our ED’s provider in triage area. His physical exam was unremarkable: he had normal vital signs; a soft and non-tender abdomen; and a normal respiratory and cardiovascular examination. His initial COWS score, performed by the treating provider, was 11, and buprenorphine 8 mg was subsequently administered. Within an hour after receiving buprenorphine, he developed restlessness, body aches, runny nose, gastrointestinal upset, anxiety, and gooseflesh skin. He did not have diaphoresis, dilated pupils, tremors, or yawning. He was moved to the main ED and was subsequently treated by an attending physician. His repeat vital signs were: blood pressure 164/88 mm Hg, heart rate 110 bpm, respiratory rate 18 breaths per minute, an oxygen saturation 98% on room air. Over the course of five hours, he was treated with ondansetron, ketorolac, lorazepam, and intravenous fluids. During his hospital course, neither blood tests nor toxicology-specific testing were performed. The patient’s repeat COWS score prior to discharge, as performed by his bedside nurse, was 6. Buprenorphine was not continued. The patient was discharged to self-care 9.2 hours after his triage time and did not follow up at the designated outpatient clinic.

All patients who received buprenorphine in the ED were discharged to home or jail. No patients were admitted to inpatient units. The median length of stay was 6.1 hours (IQR 4.7–9.0). Twenty-three (30%) patients followed up at the designated outpatient OUD clinic within one week.

## LIMITATIONS

Over the course of 18 months, the number of patients who were administered buprenorphine was relatively low compared to the number of patients who present to our ED with billing codes reflecting OUD or opioid withdrawal. We did not formally assess the barriers to buprenorphine initiation during this study period. We suspect this relatively low volume was due to the slow uptake of a novel protocol amidst the changing landscape of substance use disorder treatment in emergency medicine. Prior to the implementation of our protocol, many of our clinical staff had not heard of buprenorphine. In the first few months of the study, many of the pager calls and questions received by the EP champion were related to general buprenorphine use and to allay clinician discomfort with using the treatment protocol.

Other limitations of this study include those inherent to retrospective studies. For example, the EHR is limited to the completeness of the data recorded (eg, the time since last opioid use was not known in all cases). In addition, we reported that 16 patients received buprenorphine but did not have either a SOWS or COWS assessment performed. However, because documentation of the assessments was not compulsory in our EHR, some of these patients may have had formal assessments that were not recorded.

A final limitation was a transition from SOWS to COWS during the study period, which made it difficult to adequately compare the two. As previously mentioned, the protocol was revised to use COWS in an effort to standardize opioid withdrawal assessment in the ED and inpatient units in our hospital.

## DISCUSSION

Our study adds to the growing body of evidence regarding the feasibility of implementing a protocol to provide buprenorphine to ED patients in opioid withdrawal. As others have shown, buprenorphine remains a safe treatment option with minimal risk for precipitated withdrawal and offers an opportunity to connect these patients to ongoing addiction treatment.^11^, ^12^ In addition, we uniquely demonstrate that initiation of buprenorphine administration in the ED setting can be achieved with relatively few start-up resources: a single medical provider and pharmacist championed our protocol’s execution. As previously mentioned, D’Onofrio et al first showed the feasibility of ED-initiated buprenorphine, although they did so with the use of research associate-led interviews and referrals.^8^ Dunkley et al also conducted a retrospective review of 95 patients who received buprenorphine induction over a five-month period. While this study enrolled a large number of patients, the protocol required consultation from a specialty service, formal assessment of OUD, and admission to a clinical decision/observation unit.^12^ Therefore, although a robust protocol including consultants and specialists may lead to higher rates of buprenorphine induction in the ED setting, we demonstrate that patients with OUD may still receive adequate treatment without such resources.

Lowenstein et al studied barriers and facilitators for ED initiation of buprenorphine and showed that the largest barriers were related to patient social challenges, patient engagement to treatment, and availability of treatment referrals.^13^ Based on our study, additional barriers to the initiation of buprenorphine for OUD may be unclear chief complaint (eg, not presenting with “opioid withdrawal” or symptoms suggestive of withdrawal), inadequate screening for OUD, long ED lengths of stay, and lack of familiarity with buprenorphine or the protocol in place.

This study also underscores the safety of ED-initiated buprenorphine. Despite variations in dosing administration, most patients did not experience significant adverse events. One patient experienced precipitated withdrawal. The patient, while with an initial COWS score of 11, had last used heroin only four hours prior to ED presentation. While a formal assessment using a withdrawal scale was performed, this patient case illustrates the limitations of such scales as a screening tool. The precipitated withdrawal was most likely related to the patient’s very recent use of heroin.

In our population, 30% of patients followed up at our protocol’s designated clinic within one week of ED discharge. Our proportion of patients who attended follow-up was lower than has been seen in other studies.^8^,^11^,^18^ This can be partially attributed to other studies using an opt-in form of OUD clinic referral, selecting for patients who were more ready for pursuing treatment, rather than our referral of all-comers approach of simply providing the clinic location and instructing patients to present for follow-up on the next business day after their ED discharge.^18^ Other programs evaluated intake and retention over longer time horizons, such as 30 days, although increased lag time between time of referral and date of initial rehab intake is associated with lower rates of follow-up.^11^,^19^,^20^ So, despite less than an ideal follow-up rate in our study, our intervention still very likely led to an overall reduction in days of opioid use, which in itself has been shown to improve health outcomes.^19^,^21^ However, this potential benefit must be balanced by the fact that the time period around MOUD discontinuation is associated with increased risk of overdose death, meaning treatment initiation without retention may actually undermine benefits.^19^

## CONCLUSION

Given the magnitude of the opioid use epidemic in the United States, more formal studies of this kind are needed to demonstrate appropriate protocols for buprenorphine administration in the ED. In addition, directions for future research include the impact of the current California Bridge Program and qualitative studies to improve the rates of outpatient follow-up. It is in this way that we will be able to most adequately treat the current large proportion of vulnerable patients with opioid use disorder.

## Figures and Tables

**Table 1:** Baseline characteristics of emergency department patients who received buprenorphine for opioid withdrawal (N = 77).

Age in years (median, IQR)	37 (31–50)
Female gender	20 (26%)
Race	
Asian	1 (1%)
Black	23 (30%)
Latino/Hispanic	15 (20%)
White	37 (48%)
Unknown	1 (1%)
Chief complaint	
Opioid withdrawal, requesting detoxification	24 (31%)
Gastrointestinal upset	14 (18%)
Requesting buprenorphine	4 (5%)
Generalized pain	2 (3%)
Other	33 (43%)
Last opioid used prior to presentation	
Heroin	57 (74%)
Buprenorphine	6 (8%)
Methadone	4 (5%)
Oxycodone	4 (5%)
Other	3 (4%)
Unknown	3 (4%)
Time since last opioid use in hours (median, IQR)	
Methadone	84 (60–276)
Non-methadone opioids	24 (13–48)
ED length of stay in hours (median, IQR)	6.1 (4.7–9.0)
Withdrawal assessment	
SOWS performed	19 (25%)
COWS performed	43 (56%)
No SOWS or COWS performed	15 (19%)
Disposition	
Home or self-care	68 (88%)
Jail	9 (12%)
Follow-up at OUD clinic within 1 week	
Yes	23 (30%)

*IQR*, interquartile range; *SOWS*, short opiate withdrawal scale; *COWS*, Clinical Opiate Withdrawal Scale; *OUD*, opioid use disorder.

**Table 2:** Assessment scores and buprenorphine dose administered.

	Buprenorphine 4 mg	Buprenorphine 8 mg	No buprenorphine
SOWS			
Initial SOWS (N = 18)			
<10 (N = 0)	-	-	-
10 or above (N = 18)	1/18 (5.6%)	17/18 (94.4%)	-
2nd SOWS (N = 16)			
<10 (N = 8)	-	-	8/8 (100%)
10 or above (N = 5)	5/5 (100%)	-	-
No repeat score (N = 3)	3/3 (100%)	-	-
COWS			
Initial COWS (N = 43)			
<8 (N = 2)	1/2 (50%)	1/2 (50%)	-
8 or above (N = 39)	1/39 (2.6%)	38/39 (97.4%)	-
No initial score (N = 2)	-	2/2 (100%)	-
2nd COWS (N = 31)			
<8 (N = 12)	1/12 (8.3%)	1/12 (8.3%)	10/12 (83.3%)
8 or above (N = 15)	11/15 (73.3%)	2/15 (13.3%)	2/15 (13.3%)
No repeat score (N = 4)	2/4 (50%)	2/4 (50%)	-
Clinical judgment (or some other)			
1^st^ dose given (N = 16)	3/16 (18.75%)	13/16 (81.25%)	-
2^nd^ dose given (N = 4)	2/4 (50%)	2/4 (50%)	-

*mg*, milligram; *SOWS*, short opiate withdrawal scale; *COWS*, Clinical Opiate Withdrawal Scale.
